# Asymptomatic SARS-CoV-2 infection and CT lung lesions: how reliable is the hypothesised association?

**DOI:** 10.1259/bjro.20200068

**Published:** 2021-02-02

**Authors:** Davide Scordato

**Affiliations:** 1 Universita degli Studi di Palermo, Palermo, Italy

To the editor,

My letter concerns a recently published paper in BJR, Parry AH, et al., “Clinicoradiological course in coronavirus disease-19 (COVID-19) patients who are asymptomatic at admission”,^1^ a retrospective observational study which analysed chest CT findings in 137 asymptomatic SARS-CoV-2 positive cases admitted to an Indian hospital, and compared them to the follow-up imaging results, highlighting a plausible evolution of the aforementioned lesions among frailer patients. My letter aims to illustrate why, in my opinion, the conclusions the authors jumped to were misleading and hurried, even though further studies could likely provide better evidence for the authors’ assertions.

There are several reasons to believe this. First, the authors inferred that “asymptomatic cases with COVID-19 pneumonia have abnormal lung findings on CT”, without comparing the cases to a control group. Sampling controls from the same hospital, by applying some criteria for living areas, sex, social classes et similia, could guarantee a control group offering similar features as the case sample. On this premise, it is impossible to deduce any association if we do not take into account how cases were different from the general population living in that specific area, in terms of the prevalence of ground glass opacities (GGOs) findings. The lesions authors considered as findings in the asymptomatic cases actually lack specificity^™^. Furthermore, we should consider any possible risk factor in that area.^4^ Other causes could be responsible for such lesions to appear before the detected infection: pollution, hypersensitivity pneumonitis, some infections, malignancies, etc.

Another criticism in the study concerns the patients and imaging follow-up. Authors affirm that “the duration of hospital stay was longer in the progression group (27.1 ± 11.4 days) compared to the other three groups (16.12 ± 5.8)”. It is widely accepted that longer hospital stay is responsible for a high risk of contracting nosocomial infections.^5^ Furthermore, “the patients in progression group (54 ± 19.7 years) were older and had higher frequency of co-morbidities (46.2%) compared to the other three groups (10.4%)”, and “lower lymphocyte count”, making these patients more susceptible to nosocomial complications. Using a case-control approach would help discern whether the exacerbation of pre-existing lesions and the occurrence of new-onset lesions depended on such factors, or whether they could be attributable to SARS-Cov-2 infection.

In conclusion, even though a better methodical approach to the issue could possibly bring forth similar results, it is epistemologically and ethically incorrect to jump to such high-impact conclusions without founding these on a reliable investigation model. Deeper exploration is required in order to infer any association between an asymptomatic condition in SARS-CoV-2 positive cases and lung injury.

## Clinicoradiological course in coronavirus disease-19 (COVID-19) patients who are asymptomatic at admission: Response to letter to the editor

^1^Arshed Hussain Parry, ^2^Abdul Haseeb Wani and ^3^Naveed Nazir Shah

^1^Department of Radiodiagnosis, Sher-i-Kashmir Institute of Medical Sciences, Srinagar, Jammu & Kashmir, India

^2^Department of Radiodiagnosis, Government Medical College, Srinagar, Jammu & Kashmir, India

^3^Department of Respiratory Medicine, Chest Disease Hospital, Srinagar, Jammu & Kashmir, India

We recently described the clinical and radiological courses of an asymptomatic COVID-19 cohort wherein 44% cases had lung opacities on initial chest CT with 71% among them showing resolution of opacities and 21% revealing worsening of lung opacities on a short-term follow-up imaging (1).

Dr Davide Scordato expressed concern and wondered if in the absence of a control group, the reported ground-glass opacities (GGOs) were truly due to COVID-19 infection or were coincidental with a separate underlying cause. As mentioned in the study, ours was a dedicated COVID-19 Care Center (CCC) exclusively designed for COVID-19 cases and no other patients were admitted to the facility which made it logistically difficult to recruit a control group. Notwithstanding the availability of a control group, there are a multitude of reasons to believe that the GGOs were due to COVID-19 infection and could not be attributed to any alternate aetiology. First, GGOs are known to occur in asymptomatic COVID-19 cases. In an environmentally homogeneous cohort from the famous Diamond Princess Cruise Ship, it was reported that 54% of reverse-transcriptase polymerase chain reaction (RT-PCR) confirmed COVID-19 asymptomatic passengers and crew members had abnormal lung opacities on CT consisting mainly of GGOs.^2^ In another report of asymptomatic or minimally symptomatic COVID-19 patients from Codogno, Italy, it was found that 59% cases had bilateral lung opacities highly suspicious for COVID-19.^3^ The converse has also been reported where mildly or paucisymptomatic cases can have a normal chest CT.^4^ Second, multifocality and peripheral and basal predominant distribution of GGOs observed on CT categorized as CO-RADS-5 is typical for COVID-19 infection. In asymptomatic individuals, CO-RADS ≥3 has a low sensitivity (45%) but high specificity (89%) for diagnosing COVID-19 infection.^5^ Third, our anecdotal experiences from clinical reporting where we did not encounter such an occurrence of cluster of cases with lung opacities on CT at this time of the year when the weather is hot made us attribute the opacities confidently to COVID-19 infection. Had there been any such past experiences, we would have been careful about associating a causality relationship between the observed opacities and COVID-19 infection. Fourth, multifocality of lesions (which argues against malignancy), lack of any exposure to organic dust or animal products (which argues against hypersensitivity pneumonitis) and the absence of a correct clinical context for eosinophilic pneumonia are strong pointers to believe that GGOs were COVID-19 related. Lastly, the rapid disappearance of GGOs in a majority (71 %) of cases without any specific treatment retrospectively reinforces the infective nature of pulmonary opacities.

The second concern raised by the author was that could it be possible that the longer duration of hospital stay in deterioration group could be due to nosocomial infections. As mentioned in our study, the patients were admitted and observed in a quarantine facility of a dedicated CCC where no other patients were admitted which greatly reduces or eliminates the chances of acquiring nosocomial infections as there was no intermixing of people suffering from different infections. Also it is an established fact now that certain demographical characteristics like older age and pre-existing comorbidities protract the course of COVID-19 illness and are associated with a delayed resolution of infection.^6^

We appreciate the opportunity to address the concerns of Dr Scordato. Although GGOs are not specific to COVID-19, their occurrence in pandemic setting with a high pre-test probability, typical appearance and pattern of distribution (CO-RADS-5), rapid resolution without a specific treatment and anecdotal knowledge of population characteristics were the main reasons for attributing a causality relationship between the observed lung opacities and COVID-19 infection.
